# Predictors of Prevention Failure in College Students Participating in Two Indicated Depression Prevention Programs

**DOI:** 10.3390/ijerph110403803

**Published:** 2014-04-04

**Authors:** Vanessa Blanco, Paul Rohde, Fernando L. Vázquez, Patricia Otero

**Affiliations:** 1 Depressive Disorders Unit, University of Santiago de Compostela, Campus Vida, Santiago de Compostela 15782, Spain; E-Mail: patricia.otero@usc.es; 2 Oregon Research Institute, 1766 Millrace Drive, Eugene, OR 97403, USA; E-Mail: paulr@ori.org; 3 Department of Clinical Psychology and Psychobiology, Faculty of Psychology, University of Santiago de Compostela, Campus Vida, Santiago de Compostela 15782, Spain; E-Mail: fernandolino.vazquez@usc.es

**Keywords:** depression, prevention, risk factors, college students, classification tree analysis

## Abstract

The purpose of this study was to identify subgroups of university students with the highest likelihood of remaining at elevated levels of depressive symptoms six months following the receipt of a depressive prevention intervention on the basis of known risk factors and participation in one of two depression prevention programs. Data from a randomized controlled trial evaluating depression prevention among 133 college students with elevated depressive symptoms were analyzed. Participants were randomized to a cognitive-behavioral or relaxation training group preventive intervention. Classification tree analysis showed that older age was the strongest risk factor for persistently elevated depression. Additional risk factors were: (1) for younger students, fewer daily pleasant activities; (2) for those with higher level of pleasant activities, higher level of stressful events; and (3) for those with higher level of stressful events, lower assertiveness. Results offer directions for prevention foci, identify specific subgroups of college students to target for depression prevention efforts, and suggest that research aim to help older, non-traditional students or graduating students manage the transition from college to the work force.

## 1. Introduction

Depression is a highly prevalent mental disorder in university students, with rates of major depressive disorder (MDD) in this population ranging from 8% to 20% (e.g., [1,2,3]). Depression in college students is strongly associated with suicidal behavior, substance abuse, college dropout, loss of academic productivity, acute infectious illness, and antisocial behavior in a critical period of human development [4,5,6]. Depression during this age period has serious consequences on later occupational trajectories and an enormous economic impact over the life course [7,8].

One of the main predictors of future MDD is the occurrence of subsyndromal depressive symptoms [9], which are highly prevalent across the age span [10], with rates reaching 33% in university students [11]. These clinically significant depressive symptoms are associated with increased risk of suicidal behavior [12] and with psychosocial dysfunction and distress levels comparable to those caused by MDD [13]. For these reasons, this subgroup of individuals constitutes a relevant target population for the implementation of preventive interventions. Subthreshold symptoms of depression may consist of: (a) prodromes predicting MDD onset; (b) residual symptomatology after recovery from MDD; or (c) an independent condition, such as minor depression or recurrent brief depression, not meeting the criteria for a full-blown MDD episode [14]. Given that prevention generally refers to “interventions that occur before the initial onset of a disorder” [15] and that, by definition, the objective of prevention is to halt the first onset of a disorder [16], we excluded college students with a past history of MDD from the present trial. Targeted prevention interventions, which are focused on individuals at elevated risk, have been found to be the most effective preventive strategies [17]. Selective preventive interventions target individuals at elevated risk for depression as a function of family, environmental or personal factors; indicated prevention interventions are conducted with individuals who already show subclinical signs and symptoms of depression [18]. Few well-designed and adequately powered randomized controlled trials have evaluated the implementation of depression prevention programs in college students (e.g., [19,20]), and, to our knowledge, only one study has followed an indicated approach, comparing two indicated programs for prevention of depression in college students [21]. 

Moreover, up to date, the research on predictors in indicated prevention is limited. Some previous studies have identified variables that predict positive results of cognitive behavioral prevention and treatment programs for depression, such as younger age [22], female gender, or lower baseline level of depressive symptomatology [23]. Variables identified as depression risk factors in previous studies with college students include female gender [11], being a first year college student [24], lower social class, younger age, not being married or in a domestic partnership [25], living in urban areas [26], higher initial depression level, higher levels of irrational beliefs and stressful life events [27], and less engagement in sports activities [28]. Based on Lewinsohn’s conceptualization of depression [29,30], lower rates of pleasant activities and less assertiveness were also included as risk factors. Lastly, because of the high comorbidity between depression and anxiety [31], a measure of anxiety symptoms was included as a possible risk factor. Based on support from previous research, all of these variables at baseline were analyzed in the present study as predictors in the model. Classification Tree Analysis (CTA; [32]) is a recursive partitioning exploratory strategy used to generate hypothesis about predictors of responses on a dichotomous dependent variable. The CTA approach constitutes a nonparametric alternative to additive logistic models and has increasingly been applied to health sciences and clinical research (e.g., [33,34,35]). This procedure generates a classification tree identifying mutually exclusive subgroups whose members share similar characteristics that have high or low probability of being at risk for a dichotomous outcome, which we defined in the present study as the continued presence of elevated depressive symptoms (CES-D > 16; [36]), hence providing useful clinical information about moderation effects (*i.e.*, predicting specific subgroups who respond positively or negatively to an intervention). The identification of subgroups that respond particularly poorly or particularly well to an intervention, along with the identification of the precise cutpoints that best differentiate segments of the population at high *versus* low risk for the result (risk *vs.* non-risk), allows the definition of the target group for which the strategy will work (or not work), optimizing the use of resources in indicated prevention through the selection of specific samples that meet the characteristics that predict a good response, and developing alternative strategies for those who are less likely to benefit from a particular intervention. Among the advantages of CTA are its ability to segment populations and identify subgroups that influence the results of particular health-related behaviors [32] and its flexibility to assign the subjects in one or more steps, contrary to what happens in alternative strategies, such as discriminant or logistic models, which assign all subjects to a predefined group in a single step [37]. Moreover, when interventions are developed from regression models, they are geared toward the average subject, ignoring the special needs of population subgroups [38], and the results are difficult to interpret when three or more variables are assessed at a time [32].

To our knowledge, few studies have employed Classification Tree Analysis to examine the variables that predict the results of depression treatment (e.g., [39,40]), and only two previous studies have used this strategy to analyze the results of depression prevention interventions in the context of known depression risk factors to test whether the program effects emerge within the context of risk factors and whether the risk factors moderated program effects on risk for depressive disorder onset: one study with older female caregivers [41] and the second with high school students [42]. This is the first study using this strategy with university students.

The present study utilized data from a trial of two indicated depression prevention programs for college students. A total of 133 students with elevated depressive symptoms were randomized to a group cognitive-behavioral (CB) preventive intervention adapted from the Depression Prevention Course designed by Muñoz and Ying [43] which was derived from the empirically support treatment intervention of Lewinsohn, Muñoz, Youngren, and Zeiss [44]; or to a group relaxation training (RT) program. At 6-month follow-up, the incidence of MDD episodes in the sample was 8.6% for CB and 7.9% for RT (nonsignificant difference; total *n* = 11) [21]. The low MDD incidence rate made it a poor outcome measure for CTA. However, as previously noted, the presence of elevated depressive symptomatology constitutes one of the strongest risk factors for future MDD onset [45], which is why it has become the outcome measure in several indicated depression prevention trials, including studies in which it served as an indicator of the presence of depressive disorder (e.g., [46,47,48]). A commonly used instrument for identifying those at risk for depression has been the Center for Epidemiologic Studies Depression Scale (CES-D; [36]). In its original version, as well as in many subsequent studies (e.g., [49,50,51,52]), including university student samples (e.g., [21]), a cutoff score of 16 or greater has been used to identify individuals at elevated risk for MDD.

The aim of this study was to identify subgroups of university students who had the highest likelihood of prevention failure (*i.e.*, remaining at elevated levels of depressive symptoms) six months following the receipt of depressive prevention interventions on the basis of known risk factors and participation in two depression prevention programs. All examined predictor variables were assessed by fairly brief self-report measures, which would facilitate their use in future efforts to match students to the most appropriate prevention approach. 

## 2. Method

### 2.1. Participants

Participants were 133 university students (82% women) from Galicia, a region in the northwest of Spain, with ages ranging from 18 to 42 years (*M =* 23.3, *SD* = 4.5). All participants (100%) were Caucasian. Most were single (93%), middle-class (79%), in their first three years of academic study (56%), from urban areas (58%), and did not practice any sport (72%).

### 2.2. Procedures

Participants were recruited over two years using handbills and posters placed on campus, and advertisements in the local press, radio and television. Inclusion criteria were being a college student and scoring 16 or more on the CES-D. Exclusion criteria were current or past MDD, currently participating in other psychological or medical studies, intending to move out of the area within 9 months, or meeting criteria for other significant DSM-IV-TR Axis I disorders (e.g., dysthymia, bipolar disorders, anorexia, psychotic disorders, substance dependence, obsessive-compulsive disorder). Forty-four of the initial 177 participants (25%) did not meet the inclusion/exclusion criteria or declined participation upon learning the nature of the study. The remaining 133 participants were assigned using a random numbers table to either (a) CB group (*n* = 70) or (b) RT group (*n* = 63) by a statistician with no other role in this study (for further details and rationale about RT being used as a strategy for the prevention of depression, see [21]). Participants were assessed by two trained PhD level psychologists, blinded to preventive intervention condition, at pretest, posttest, 3-, and 6-month follow-ups. The sociodemographic, academic and clinical data were obtained through a clinical interview and a series of self-administered questionnaires (for further detail, see the Measures section), with a typical duration of 90 min. Participation was voluntary, with no economic or other kinds of incentives. Two therapists administered both conditions, and groups were led by a single therapist. Therapists in both conditions were trained specifically for the study in eight 90-min seminars led by two clinicians with expertise in cognitive-behavioral and relaxation methods. Additionally, in a pilot study conducted prior to the present investigation, each therapist conducted two groups, with 5–6 participants per group. All therapy sessions were videorecorded and viewed by one of the training clinicians to assess therapist adherence to the prevention protocols and therapist skillfulness using established measures. The study was approved by the ethics committee of the University of Santiago de Compostela. All participants gave written informed consent.

### 2.3. Prevention Conditions

Group CB was adapted from the Depression Prevention Course designed by Muñoz and Ying [43], originated in the works of Lewinsohn *et al.* [44]. This group focused on managing negative thoughts, increasing pleasant activities, and training in assertiveness and social skills. This program has shown efficacy in previous randomized trials [53,54]. RT focused on progressive muscular relaxation [55], breathing control, visualization, and meditation [56]. In a review of randomized clinical trials that used relaxation procedures in patients with clinical depression [57], muscle relaxation training was more effective in the decrease of patient self-rated depression than minimal treatment or nontreatment. These encouraging results in clinical population suggest that it could be useful for those experiencing significant depressive symptoms. However, to the date, relaxation training has not been used in randomized clinical trials of prevention of depression. Both prevention interventions consisted of eight weekly 90-min sessions administered to groups of 5–6 participants by two therapists, each with four years of experience conducting psychotherapy.

### 2.4. Measures

*Demographic, academic and health factors* included gender, age, marital status, high/medium/low social class (categorized as high, medium, low), academic year, rural/urban residence, and engagement in sports activity; measures were evaluated by a questionnaire specifically developed for this study. 

*Major depression diagnosis* was assessed by trained psychologists with the Structured Clinical Interview for DSM-IV Axis I Disorders, Clinician Version (SCID-CV; [58]), whose interrater reliability (kappa) ranges from 0.70 and 1.00. 

*Depressive symptoms* were evaluated using the Center for Epidemiologic Studies Depression Scale (CES-D; [36]), a 20 four-point item scale (ranging from 0 = *rarely or none of the time*, to 3 = *most or all of the time*) whose internal consistency ranges from 0.85 to 0.90 [59]. It includes four factors: (a) somatic disorders; (b) depressive affect; (c) positive affect; (d) interpersonal problems. Higher scores indicate higher symptomatology. A score of 16 or higher suggests clinically significant depressive symptomatology. Alpha for the present study at baseline was 0.81.

*Anxiety symptoms* were assessed using the Beck Anxiety Inventory (BAI; [60]), a 21 four-point item scale (ranging from 0 = *none* to 3 = *severe*) that discriminates between anxiety and depression; the total score ranges from 0 to 62, with higher scores indicating higher anxiety symptomatology; according to the authors, internal consistency at baseline ranges from 0.90 to 0.94, and test-retest reliability from 0.70 to 0.93. Alpha for the present study at baseline was 0.85.

*Irrational beliefs* were evaluated using the Belief Scale (BS; [61]), a 20 five-point item scale (ranging from 1= *totally disagree* to 5= *totally agree*) measuring irrational beliefs as conceptualized in rational-emotive behavior therapy, whose alpha was 0.80, with a 2-week test-retest reliability of 0.89 [61]. In a study evaluating its construct validity, the authors found a correlation with the Irrational Beliefs Test (IBT; [62]) of 0.55, and in a study evaluating its discriminant validity, a correlation of −0.27 with the Social Desirability Scale [63]. Higher scores indicate a higher level of irrational beliefs. Alpha for the present study at baseline was 0.75.

*Assertiveness* was evaluated with the Rathus Assertiveness Schedule (RAS; [64]), a 30 six-point item scale (ranging from −3 = *not at all characteristic of me*, to +3 = *very characteristic of me)*. The score ranges from −90 to +90, with lower scores indicating non-assertiveness, and higher scores indicating higher assertiveness. The original version was shown to have moderate to high test-retest reliability (*r* = 0.78; *p <* 0.01). Alpha for the present study at baseline was 0.83.

*Number of pleasant activities* was evaluated using the Pleasant Activities Checklist [65], a 100 item dichotomous response (yes/no) checklist that measures the number of daily pleasant activities. Higher scores indicated a higher rate of engagement in pleasant activities. Alpha for the present study at baseline was 0.91.

*Life events* were assessed using the Recent Life Changes Questionnaire (RLCQ; [66,67]), an 87 item dichotomous response (yes/no) checklist that covers stressful events that occurred in the last year, with each event assigned a specific value of Life Change Units. The final value is the sum of Life Change Units for all reported stressful events. Higher scores indicated more stressful events in the rated period. According to the authors, 3-week test-retest reliability was 0.85. Alpha for the present study at baseline was 0.72. 

The original measures were translated for the study, following published guidelines [68], which include the translation/back-translation method [69].

### 2.5. Data Completeness and Data Analysis

Attrition in the study was low: 95% of participants completed posttest assessment, 93% completed 3-month follow-up, and 93% completed 6-month follow-up. An intention to treat analysis was conducted using the last observation carried forward technique. A polychoric correlation matrix was performed using STATA 12, as it allows for the computation of associations between different combinations of continuous, ordinal, and dichotomous variables. 

Classification Tree Analysis (CTA) was conducted using SPSS Statistics 19. CTA identifies subgroups whose members share similar characteristics that influence the dependent variable, in our case, elevated depressive symptoms (CES-D > 16) 6 months following receipt of a prevention intervention. The growing method was Classification and Regression Trees (CRT), which splits the data into segments (*i.e.*, branches of the tree) as homogeneous as possible with respect to the dependent variable. The splitting criterion was the Gini improvement measure [32]. Larger values indicate greater difference with respect to the prevalence of the dependent measure in the two child nodes (*i.e.*, branches made from a parent node). Once all cases in a branch have approximately the same probability for the dependent variable, it is considered a “terminal node”. Three stopping rules were determined a priori: (1) the minimum number of cases in the parent and child nodes could be no smaller than 20 and 10, respectively, as established for previous studies analyzing the results in randomized clinical trials [42]; (2) the maximum number of levels to which the tree could grow was 5 (the default value for CRT); and (3) the minimum value of the Gini improvement measure could not be smaller than 0.001, which indicates modest differences between two nodes [32]. When the sample size is too small for independent cross-validation, it has been recommended to use sample subgroups for cross-validation [70]. A tenfold cross-validation procedure was conducted to determine the optimal tree size and structure, for which the sample was divided into ten groups (each containing 10% of the sample), and ten trees were constructed, each leaving out a different 10%. The final tree was the one showing the best average accuracy for cross-validated predicted classification. Odds ratio and 95% confidence intervals were calculated by logistic regression. 

To evaluate the classification accuracy of the CTA model as branches were added to the tree, we computed sensitivity (*i.e.*, proportion of individuals identified by the model as high-risk among the total sample of high-risk individuals), specificity (*i.e.*, proportion of individuals identified by the model as low-risk among the total sample of low-risk individuals), positive predictive value (PPV; proportion of true high-risk individuals among those identified by the model as high-risk), and negative predictive value (NPV; proportion of true low-risk individuals among those identified by the model as low-risk) after each model split.

## 3. Results

The CB and RT groups did not differ significantly either in regard to levels of risk on the depression screener or on risk factor variables at baseline.

### 3.1. Risk for Depression Persistence

The rate of persistent elevated depressive symptoms (*i.e.*, elevated CES-D score post-intervention) was significantly higher in the RT group than in the CB group by posttest (60% *vs.* 37%, respectively); χ^2^ (1, *N* = 133) = 7.13, *p* = 0.008. By 6-month follow-up, 71 (53%) of the participants still had elevated depressive symptom levels, 54% in the CB condition and 52% in RT, χ^2^ (1, *N* = 133) = 0.05, *p* = 0.826. 

### 3.2. Risk Factor Variables

Table 1 contains the intercorrelation matrix for the 13 risk factors at baseline and the correlations between each risk factor and the outcome measure of persistent elevated depressive symptoms. Also shown are means and standard deviations for continuous measures and percent occurrence for dichotomous measures. The strongest associations among risk factors occurred between depressive symptoms and anxiety symptoms (*r* = 0.57), gender and academic year (*r* = −0.37) (being a male and higher academic year significantly correlated), marital status and academic year (*r* = 0.36) (being single and lower academic year significantly correlated), age and irrational beliefs (*r* = −0.35), and anxiety and assertiveness (*r* = 0.35). Five of these variables were significantly correlated with the outcome measure. Assertiveness at pretest had the largest correlation with risk for future elevated depression (*r* = −0.36), followed by age (*r* = 0.27), anxiety symptoms (*r* = 0.26), social class (*r* = 0.26), and number of pleasant activities (*r* = −0.24). 

**Table 1:** Intercorrelation matrix of examined risk factors.

Variables	1	2	3	4	5	6	7	8	9	10	11	12	13
1. Male sex	1												
2. Age	*−0.216*	1											
3. Single marital status	−0.029	*0.177*	1										
4. High social class	0.234	0.040	−0.036	1									
5. Academic year	**−0.369**	*0.213*	**0.357**	*−0.189*	1								
6. Rural residence	−0.156	0.266	0.044	−0.023	*0.200*	1							
7. Sport practice	0.145	−0.098	−0.234	−0.102	−0.029	*−0.181*	1						
8. Anxiety symptoms	0.112	0.259	0.271	−0.009	0.224	0.144	*0.173*	1					
9. Depressive symptoms	0.141	0.076	0.073	−0.123	0.149	0.117	0.166	**0.571**	1				
10. Irrational beliefs	−0.146	**−0.354**	0.129	−0.055	0.136	−0.035	−0.023	0.119	0.241	1			
11. Assertiveness	0.036	0.244	−0.146	0.132	−0.165	0.093	−0.068	**−0.354**	*−0.216*	**−0.280**	1		
12. Pleasant activities	**−0.325**	0.082	−0.069	−0.145	−0.046	*0.179*	−0.017	−0.131	−0.232	*−0.181*	*0.175*	1	
13. Life change units	0.162	**0.291**	0.041	0.048	−0.121	*−0.*025	−0.029	0.072	0.001	−0.067	0.240	0.055	1
Mean (% occurrence for 1, 3, 4, 5, 6, 7)	18.0	23.3	93.2	7.5	55.6	41.4	27.8	15.0	25.3	69.6	−14.1	17.5	382.9
Standard deviation	NA	4.5	NA	NA	NA	NA	NA	8.5	7.9	8.6	20.4	10.8	207.1
Correlation with outcome	0.032	0.272	0.012	0.256	**0.163**	0.011	0.093	0.261	0.154	−0.022	**−0.359**	−0.238	0.105

Note: All the variables were assessed at baseline. Italicized correlations are significant at *p* < 0.05, underlined correlations at *p* < 0.01, bold correlations at *p* < 0.001.

### 3.3. Classification Tree Analysis

**Figure 1:**
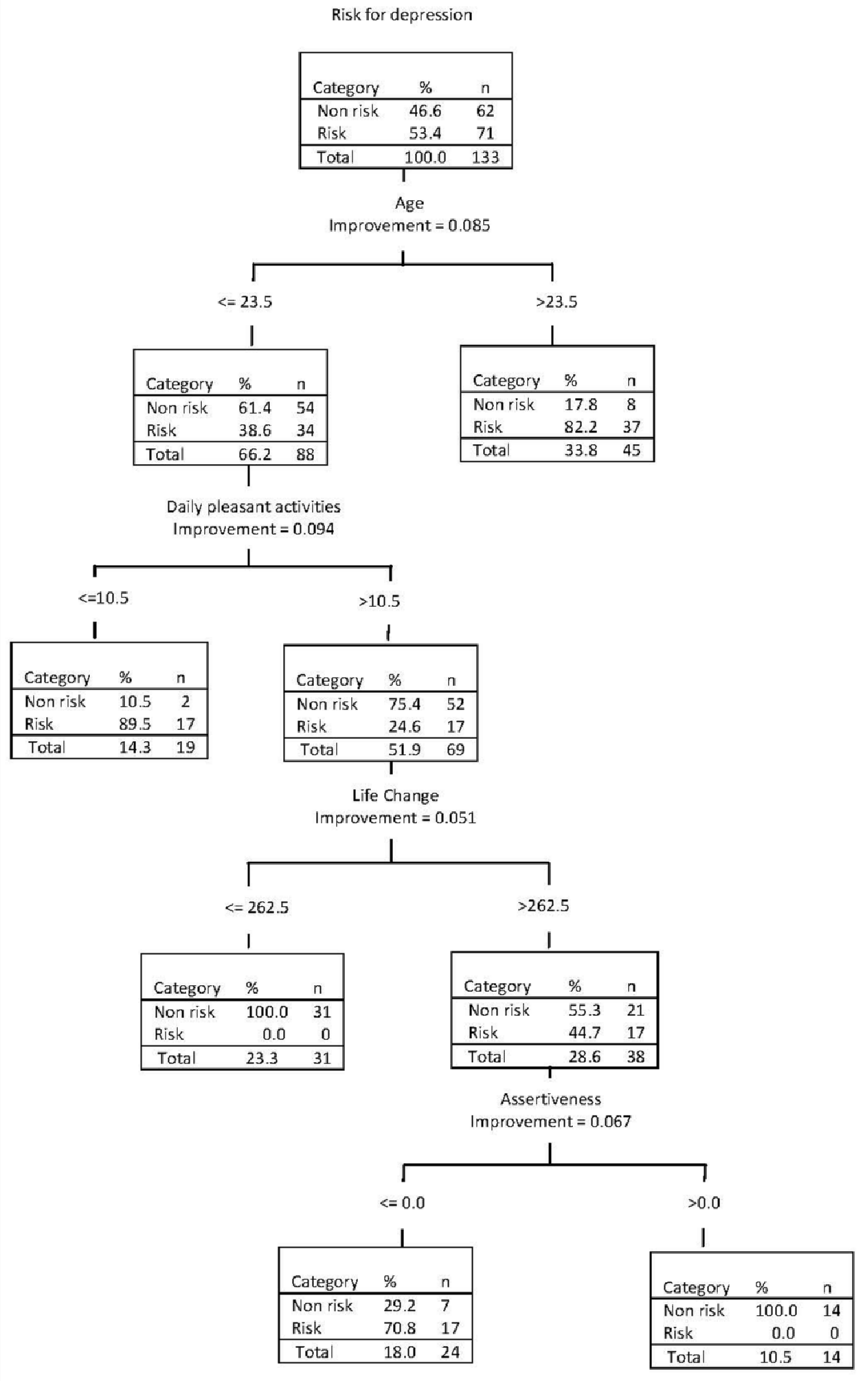
CTA decision rules predicting risk of persistent depression with pretest variables and prevention conditions by 6 month follow-up.

Prevention condition (1 = CB, 2 = RT) and risk factors at baseline were entered into a CTA with tenfold cross-validation, producing a classification tree with four forks and five terminal nodes (see Figure 1). The first fork consisted of age, which emerged as the most potent predictor of risk for persistent depression. The rates of elevated depressive symptoms among university students who were older than 23.5 years were more than double that for young students (82% *vs.* 39%; Gini = 0.085; *OR* = 7.35, 95% *CI* = 3.06–17.65). The first fork correctly identified 52% of the cases (sensitivity) and 87% of the non-risk cases (specificity). Of the predicted cases, 82% were true cases (PPV) and 61% of the predicted non-cases were true non-cases (NPV). 

Among college students who were younger in age, an additional branch emerged. Participants with a lower number of pleasant activities (≤10.5) were at a threefold increased risk for persistent elevated depression levels compared to those reporting higher rates of pleasant activities (89% *vs.* 25%; Gini = 0.094; *OR* = 10.61, 95% *CI* = 3.47–32.49). This second fork correctly identified 76% of cases (sensitivity) and 84% of non-cases (specificity); PPV = 84% and NPV = 75%.

Among the subgroup of younger university students with higher number of pleasant activities, an additional branch emerged. High rates of stressful life events (RLCQ > 262.5) at pretest significantly predicted risk for depression (45% *vs.* 0%, Gini = 0.051; *OR* = 1.81, 95% *CI* = 1.36–2.41). The addition of this third fork improved correct identification of cases (sensitivity = 100%) and correctly identified 50% of the non-cases (specificity); PPV = 70% and NPV = 100%. 

One last branch, assertiveness, emerged for the younger college students with both higher pleasant activity levels and life change units. Lower, compared to higher, assertiveness emerged as a predictor of risk for persistent elevated depressive symptoms (71% *vs.* 0%, Gini = 0.067; *OR* = 3.43, 95% *CI* = 1.84–6.60). This fourth fork correctly identified 100% of cases (sensitivity) and 73% of non-cases (specificity); PPV = 81% and NPV= 100%. The final CTA model correctly classified (accuracy rate) 87% of the total sample (to examine CES-D scores as a continuous rather than dichotomous measure, we conducted an exploratory regression analysis predicting CES-D scores at 6-month follow-up. The regression was restricted to variables that had a univariate association of *p* < 0.20. In the final multiple regression analysis, the same four baseline variables selected in the CTA model emerged as significant predictors (*i.e.*, age, number of pleasant events, stressful events, and assertiveness), in addition to experimental condition, marital status, social class, and depressive symptoms).

## 4. Discussion

The primary aim of this study was to identify subgroups of college students with the highest likelihood of prevention failure, which we defined as remaining at elevated levels of depressive symptoms by 6-month follow-up, on the basis of known depression risk factors and participation in two depression prevention programs. 

By 6-month follow-up, 53% of the participants still had elevated depressive symptom levels, 54% in the CB condition and 52% in RT, with no differences between groups. The fact that a relaxation training intervention achieved comparable results to a well-supported intervention, such as the CB condition, could be explained through specific mechanisms underlying relaxation, such as anxiety reduction [57], or could be due to an increment in self-efficacy, as suggested by Stice *et al.* [47] in line with the proposal of Bandura [71] of self-efficacy as a process central to therapeutic change.

Older age emerged as the most important risk factor in this sample for persistently elevated depressive symptoms. Participants who were older than 23.5 years of age (34% of the sample) were at high risk for remaining at elevated depression levels by 6 months post-intervention, having twice the probability of elevated symptoms as students who were under 23.5 years of age. This finding contrasts with previous studies in this population, which found that younger age [25] or being a first year college students [24] constituted risk factors for depression. A further look at the present results shows that age was significantly correlated to academic year, anxiety symptoms, and life change units, suggesting that increased stress and anxiety could be related to the high risk for depression in the older age group. One possible hypothesis is that college students who are nearing graduation could be experiencing more stress, both academic and related to future life plans, which would account for their risk of persistently elevated depression levels. A previous study [72] found an increasing amount of stress in last-year medical students, related to depression and anxiety. However, the fact that age and stress (*r* = 0.29) and age and anxiety (*r* = 0.26) were significantly positively correlated in the present study may explain why age was only selected as a predictor for the whole sample and does not imply that stressful situations or anxiety are not general depression predictors but rather that their effects overlap with age. Other possible hypotheses to explain this role of age is that non-traditional students who either entered college at an older age or took longer to complete college, possibly due to having to work or other responsibilities, such as parenting, while also attending university experience higher levels of stress or lower social support, which maintained elevated depressive symptom levels. 

For the majority of the sample that consisted of younger college students, the most important predictor for remaining at elevated risk for depression was engaging in a low number of daily pleasant activities. This finding supports Lewinsohn’s conceptualization of depression [29,30] and reinforces the recommendation of activity scheduling as an efficacious, straightforward, and time-efficient approach for managing depression and subthreshold depressive symptoms in this age group [73,74]. Interestingly, the cutoff point for risk of persistent depression selected by the CTA model to maximize prediction was approximately 10% of the available activities per day on this version of the PES (10.5 activities out of 100 options); this rate is very close to the mean activity level for nondepressed adults (31.9 out of 320 possible activities on the original PES; [75]) and a higher rate of behavioral activation than found in depressed adults [75,76]. However, the diversity of measurement instruments and contextual factors makes it difficult to directly compare the specific cutpoints for various studies. A question that remains unresolved is why this variable emerged as a predictor only for the younger students. A tentative explanation is that the older students are facing more immediate issues as they transition to adult lives, where the achievement of developmental goals related to getting a job [77], housing, or parenthood could be more influential in student well-being than the current rate of engagement in pleasant activities. The fact that this variable emerged as the strongest predictor of persistently elevated depression levels among the majority of college students provides strong support for behavioral activation interventions to prevent MDD onset in this relatively high-risk population.

For students who were engaging in a high number of pleasant activities, the presence of stressful life situations emerged as the next risk factor for continued depression. Interestingly, none of the students with high behavioral activation levels and low stressful life events remained at elevated depression levels. All participants in this study received one of two depression prevention interventions; we do not know if this subgroup of students with high behavioral activation/low stress would have experienced reductions in their depressive symptom levels simply with the passage of time. The selection of this branch is consistent with a study of Australian college students, which found that stressful life events were predictive of elevated depressive symptoms at the end of the academic year [27] and with a study of Chinese medical students, which found a correlation between stressful events and mental health problems that was moderated by resilience [78]. A previous study with younger (high school) students [79] found that stress moderated the impact of a CB group preventive intervention relative to assessment control: at lower levels of negative life events, the CB group had significantly reduced depressive symptoms levels compared to assessment control, but for students with high levels of stress, the CB prevention program did not decrease depressive symptoms compared to controls. Another study [80] found that self-reported sources of stress in students at a mid-sized, Midwestern US university varied as a function of year in school, age, gender, and academic major, and were associated with changes in sleeping habits, vacations/breaks, eating habits, and increased workload and new responsibilities; all of these associations with stress during college could account for the present findings. 

Finally, for the college students who reported high levels of fun activities but also high stress, their level of assertiveness made the difference between remaining at elevated depressive levels or not. As with engagement in pleasant activities, this finding supports Lewinsohn’s model of depression [29,30] and is consistent with previous studies which found low assertiveness to be a risk factor for depression [81,82]. Interestingly, the cutoff score for this scale that was statistically selected by the CTA approach was zero, which constitutes the point separating negative and positive assertiveness. These findings are consistent with Lazarus and Folkman’s Stress and Coping Model [83], which hypothesizes that under stress, individuals make two appraisals: the primary appraisal, which is an assessment of how stressful or threatening the situation is; and the secondary appraisal, which is an assessment of one’s ability to cope. Perhaps college students with high assertiveness evaluate potentially stressful situations as a challenge, as suggested by previous research [84] or are more confident about their capabilities to cope with these stressful situations and achieve good results. 

In the previous study that used CTA to predict MDD onset among high school students on the basis of known risk factors and involvement in various depression prevention interventions [42], different variables emerged as risk factors. Negative attributional style emerged as the most important risk factor for MDD; for those with high negative attributional style, prevention condition (bibliotherapy) emerged as the main predictor as a protective factor; for those with low negative attributional style, depressive symptoms emerged as the most important risk factor; noteworthy is that negative life events failed to emerge as a predictor in CTA. The failure to achieve similar predictors could be due to several factors. First, the present design included two active conditions which may have made it more difficult for prevention condition to emerge as a risk factor as all participants tended to benefit; the earlier trial compared two active interventions to or a non-active control. On a related note, participants showed low disorder incidence rates after the intervention, which meant that MDD incidence could not serve as the outcome measure in the present study, as was done in the previous CTA trials. More importantly, each study included unique risk factor variables, in addition to common depression risk factors. The fact that some of the same risk factors were analyzed in multiple studies but emerged as predictors only for one population (e.g., life events emerged as a risk factor for this study, but not for Rohde *et al.* [42]) highlights the needs to take a developmental and contextual perspective and to analyze specific predictors for diverse populations in preventing depression. It should also be noted that the failure of an examined risk factor variable to be selected as a branching variable in the CTA tree should not be taken as evidence that the factor is not predictive; instead it may suggest that the predictive effects of the measures were weaker in magnitude than the selected risk factors or were masked by other correlated variables.

It is important to note the limitations of this study. First, the relatively small sample size resulted in limited power to detect moderation effects and an inability to independently replicate the classification model. Though independent cross-validation was not possible because of the sample size, we conducted a tenfold cross-validation procedure to determine the optimal structure of the tree. Second, the low number of participants who developed MDD during the course of the study prevented us from using MDD onset as the outcome measure. We chose instead to focus on identifying predictors of elevated depressive symptoms (*i.e.*, prevention failure), which is consistently found to be one of the strongest predictors of MDD onset. Third, it was noteworthy that preventive intervention condition failed to emerge as a predictor in this sample and it is possible that the mechanisms of change for the CB and RT interventions were too similar to be detected with the present level of statistical power to result in different predictive effects in the CTA model. The inclusion of a placebo prevention intervention or a no-intervention control condition may have provided an opportunity to detect differential effects of the prevention programs. Fourth, other relevant depression risk factors were not included in this study, such as past history of MDD, other psychiatric disorders, and family history of depression. Our goal in this study was to examine the predictive effects of risk factors that could be easily and accurately obtained from the student self-report. On a related note, the decision to exclude students with past MDD and those with current diagnoses of other major DSM-IV-TR Axis I disorder was intended to provide strong internal validity to the evaluation of the two depression prevention interventions, but limited the generalizability of findings. A last noted limitation is the absence of a longer follow-up period; however, we were particularly interested in the potential effects of two prevention interventions in reducing depressive symptoms levels and a review of the literature in this area indicates the fairly rapid erosion of intervention effects, which begins to occur shortly after the end of any intervention [18]. Future trials comparing the two active preventive interventions to each other and possibly to standard care with a larger and possibly more inclusive sample, to better detect potential moderation effects, would be highly informative. Also, trials comparing the active interventions to untreated controls would provide a method of distinguishing prevention intervention effects from the natural course of depressive symptom persistence and depressive disorder onset.

## 5. Conclusions

In summary, the present results found that: (1) being over 23 years of age while attending university confers the highest discriminative risk for failing to benefit from an indicated depression prevention intervention; (2) for younger students, the risk for persistently elevated depression levels increases if they are not engaged in an adequate number of pleasant activities; (3) among students who are engaging in pleasant activities, in the present of stressful life circumstances, almost half will remain persistently depressed; and (4) for those exposed to high stress, low assertiveness predicts a higher risk for remaining depression, while high assertiveness appears to act as a protective factor.

These findings have potentially important implications for both research and clinical practice. Although the stress of entering college is well-recognized (e.g., [85]), the current finding that older students who received a depression prevention intervention were at the greatest risk for remaining at elevated levels of depressive symptoms suggests the need to help graduating students successfully manage their transition from college into the work force and to help the older, non-traditional student (10% of the sample was 29 years of age or older at baseline). The findings that lack of engagement in pleasant activities and low assertiveness increase the likelihood of persistent for depression in the challenging lives of college students provides additional empirical support for the behavioral model of depression proposed by Lewinsohn and colleagues more than 35 years ago and provides clinicians with useful information for the development of specific preventive interventions whose strategies are tailored to the diverse needs of subgroups of university students. In general, given their high risk for elevated depressive symptoms and MDD, more research is needed on preventing depressive conditions among the population of college students. Our hope is that the present study will offer some suggestions for specific high-risk subgroups to target and potential foci for most effectively intervening.
